# Heat Transfer in Biological Spherical Tissues during Hyperthermia of Magnetoma

**DOI:** 10.3390/biology10121259

**Published:** 2021-12-02

**Authors:** Mahmoud Ragab, Ahmed E. Abouelregal, Huda F. AlShaibi, Rasha A. Mansouri

**Affiliations:** 1 Information Technology Department, Faculty of Computing and Information Technology, King Abdulaziz University, Jeddah 21589, Saudi Arabia; 2 Centre of Artificial Intelligence for Precision Medicines, King Abdulaziz University, Jeddah 21589, Saudi Arabia; 3 Department of Mathematics, Faculty of Science, Al-Azhar University, Nasr City, Cairo 11884, Egypt; 4 Department of Mathematics, College of Science and Arts, Jouf University, Al-Qurayyat 75911, Saudi Arabia; ahabogal@mans.edu.eg; 5 Basic Sciences Research Unit, Jouf University, Sakaka 2014, Saudi Arabia; 6 Department of Mathematics, Faculty of Science, Mansoura University, Mansoura 35516, Egypt; 7 Department of Biochemistry, Faculty of Sciences, King Abdulaziz University, Jeddah 21589, Saudi Arabia; halshaibi@kau.edu.sa (H.F.A.); amansouri@kau.edu.sa (R.A.M.)

**Keywords:** bioheat transfer, hyperthermia, MGT Pennes equation, prediction, spherical tissues

## Abstract

**Simple Summary:**

Heat transport in biological tissue is mediated through a variety of phenomenological processes, involving tissue heat exchange, blood-tissue convection, blood perfusion or advection and diffusion across microvascular beds, and metabolic heat production. In recent years, many physicians and engineers have taken an interest in applying computational and mathematical techniques to model biological systems. The objective of the current paper is to provide an analytical solution to the modified Pennes bioheat conduction equation with a single relaxation time. The suggested model is used to examine heat transport in biological tissues as an infinite concentric spherical region during magnetic fluid hyperthermia. This method is used to investigate the influence of heat generation through heat treatment on a skin tumor a spherical layered structure. The present model can explain the effect of different therapeutic approaches such as cryotherapy sessions, laser therapy, and physical occurrences including transfer, metabolism support, blood perfusion, and other similar treatments.

**Abstract:**

Hyperthermia therapy is now being used to treat cancer. However, understanding the pattern of temperature increase in biological tissues during hyperthermia treatment is essential. In recent years, many physicians and engineers have studied the use of computational and mathematical models of heat transfer in biological systems. The rapid progress in computing technology has intrigued many researchers. Many medical procedures also use engineering techniques and mathematical modeling to ensure their safety and assess the risks involved. One such model is the modified Pennes bioheat conduction equation. This paper provides an analytical solution to the modified Pennes bioheat conduction equation with a single relaxation time by incorporating in it the (MGT) equation. The suggested model examines heat transport in biological tissues as forming an infinite concentric spherical region during magnetic fluid hyperthermia. To investigate thermal reactions caused by temperature shock, specifically the influence of heat generation through heat treatment on a skin tumor [AEGP9], the Laplace transformation, and numerical inverse transformation methods are used. This model was able to explain the effects of different therapeutic approaches such as cryotherapy sessions, laser therapy, and physical occurrences, transfer, metabolism support, and blood perfusion. Comparison of the numerical results of the suggested model with those in the literature confirmed the validity of the model’s numerical results.

## 1. Introduction

Hyperthermia is a highly interesting topic in medicine. Several studies have been conducted on the use of heat transmission to living tissues for cancer treatment [1] as well as to enhance treatment procedures and create more sophisticated and precise technologies for forecasting temperature in biological tissues. Such studies have led to the development and use of hyperthermia therapy, also known as “thermal medicine” or “thermotherapy,” a kind of treatment in which the body’s immunity and ability to self-heal are stimulated by exposing the body to high temperatures. Hyperthermia can be used to treat a specific area of the body or the entire body. It is used in conjunction with standard treatments and is available only through referral and under the supervision of a healthcare practitioner [2].

In recent years, hyperthermia therapy has been shown to be useful in treating cancer. Its goal is to increase the heat of diseased tissues to beyond cytotoxic levels (41 °C to 45 °C) while avoiding overexposure of healthy tissues [3,4]. In the treatment of some kinds of cancer, like liver metastases, radiation combined with traditional hyperthermia is more successful than radiation alone [5].

Hyperthermia nowadays is a highly interesting topic in medicine. Several studies have been conducted on the use of heat transmission into living tissues, particularly for cancer treatment [1]. In addition, various studies have been applied over the years as well as to enhance treatment procedures and build create new more sophisticated, and precise technologies, with the goal of forecasting temperature in biological tissues. One hyperthermia technique for treating tumors is magnetic fluid hyperthermia (MFH). It is a non-invasive approach in which magnetic nanoparticles are injected into the tumor as heat mediators, after which the tumor is exposed to an external alternating magnetic field (AMF) [6]. MFH has various advantages over NIR laser-based hyperthermia. Its magnetic targeting method for cancer therapy achieves greater magnetic field penetration into tissues and increased accumulation of magnetic nanoparticles in tumors [7]. Its most important advantages are that magnetic fluid hyperthermia-based multimodal cancer treatments are more successful for cancer treatment due to their synergistic action, particularly when combined with chemotherapy [7].

Hyperthermia treatment can also be performed using a radiofrequency (RF) generator with electrodes and antennas [8], as well as ultrasonic, microwave, and laser irradiance [9].

These diverse techniques highlight the complexity of heat transmission in biological systems because many physiological functions rely on the spatiotemporal temperature differences in live biological tissues, and heat transmission in biological systems involves a variety of mechanisms that facilitate heat transmission in biological tissues and that must be considered, including convection between the blood and tissues, heat transfer in tissues, blood perfusion or delayed blood perfusion, the vascular structure, diffusion through microvascular beds, metabolic heat generation, and changes in tissue properties depending on physiological conditions, among others [5,10,11]. Thus, the success of the treatment is determined not only by the technology used but also by a thorough examination of the complex bioheat transfer process and the pattern of temperature increase in biological tissues during hyperthermia treatment [12]. Hyperthermia treatment can also be done performed using a radiofrequency (RF) generator with electrodes and antennas [8]. In other words, the issue of heat transmission in biological systems is complex because it involves a variety of mechanisms to consider, including convection, heat transfer in tissues, perfusion of blood, the vascular structure, metabolic heat generation, and changes in tissue properties depending on physiological conditions, among others [1]. An accurate explanation of the thermal relationships between blood vessels and tissues is required for the use of medical technology to be used in the treatment of deadly diseases such as cancer. To provide a therapeutic temperature while avoiding overheating and damage to surrounding healthy tissue, the temperature distribution within and outside the target area must be understood as a function of the exposure duration [3,4].

The study of temporal and geographical variations in temperature is required while investigating bioheat transportation concerns because many physiological functions rely on the spatiotemporal temperature differences in live biological tissues. Many biologists, physicians, mathematicians, and engineers have developed mathematical models of heat transfer in biological tissues Mathematical models are now often used in the study of hyperthermia in tumor treatment, oncology, cryosurgery, and many other applications [13]. There have been several models developed to regulate such heat transfer [14,15,16].

An empirical law of thermal conductivity known as “Fourier’s law” describes heat transfer in a continuous medium. Unfortunately, since the Fourier formula produces an infinite speed of heat spread due to the parabolic form of the diffusion equation, it has not been used in cases of fast transient heat transfer such as pulsed laser irradiation. As a result, a finite heat diffusion velocity must be determined. The Pennes model of bioheat transfer is widely used in modern engineering and medical therapy because of its simplicity [14], although it must be adjusted to account for the particular characteristics of the tissue under investigation. It is based on the Fourier equation for thermal conductivity, which predicts the propagation of thermal disturbances at an infinite rate. As a result, a restricted heat transfer rate must be established.

The Pennes bioheat equation incorporates the effects of diffusion, advection, volumetric heat production from metabolism, and spatial heating on heat transfer in a biological organism. The thermophysical characteristics of tissues, such as thermal conductivity, density, and specific heat, determine the diffusion and transitory thermal impacts [17,18].

According to researchers [19,20], Pennes’ explanation of the vascular contribution to heat transmission in perfused tissues fails to explain the real thermal equilibration process between streams of flowing blood. Consequently, for effective hyperthermia treatment, accurate thermal modeling is required. Khanafer et al. [21] used physiological velocity waveforms to compute and analyze how the pulsatile laminar flow and heating protocol affect temperature variation in a single blood artery and tumor tissue that are undergoing hyperthermia therapy. They found that the existence of big vessels has an important impact on temperature variations, which must be considered when planning hyperthermia therapy [22]. They further found that a uniform heating system has a wider temperature spread than the pulsed heating scheme, which may cause overheating in areas that may damage normal tissues [13].

Several computational and experimental approaches have been developed to solve the biothermal equation since it is critical to make an accurate calculation of the temperature range across the entire affected region. Kundu [23] used the variable separation method to express exactly the temperature sensitivity in biological tissues based on the Fourier and non-Fourier heat transfer conditions during therapeutic settings. Kumar et al. [24] studied the dual-phase-lag (DPL) concept of bioheat transport with a Gaussian distribution source term under the most generalized boundary condition during hyperthermia treatment. To approximate an analytical solution to the current problem, the finite element wavelet Galerkin approach, which uses the Legendre wavelet as a basic function, was employed. Liu et al. [25] developed the bioheat transfer equation based on the DPL model to address the effect of microstructural interaction. They investigated the bioheat transfer problem in the skin, which was considered a three-layer composite, using the appropriate equation. Lin and Li [10] proposed an analytical solution to bioheat transport in skin tissue with broad boundary conditions using the Pennes, Cattaneo–Vernotte, and DPL models. They looked at the heat transfer of skin that has been subjected to pulse laser heating and fluid cooling.

Jaunich et al. [26] investigated the temperature change and the heat-affected area after treating a skin tissue medium with a collimated or focused laser beam from a pulsed laser source. Experiments were conducted on multilayer tissue phantoms that resembled skin tissue, and on freshly excised mouse skin tissue samples with implanted heterogeneities that simulated subsurface tumors. Maamoun et al. [27] described their use of microwave antennas for microwave imaging of tumors inside the liver and predicted the temperature profile in the liver and inside and outside the tumor throughout hyperthermia with and without nanoparticles, using a computer simulation of a genuine human model. Majchrzak and Stryczyski [28] investigated the heat transmission between blood vessels and biological tissue using the DPL theory.

The impact of the heating method is described by maintaining a consistent temperature for the tumor that is higher than the blood and tissue temperatures. To evaluate a local thermal non-equilibrium (LTNE) bioheat model, Dutta and Kundu [29] presented an analytical hybrid scheme that consisted of a shift of variables and a finite integral transform. This system may be used to improve transient temperature prediction in the treatment of cancer patients using localized hyperthermia treatment (LHT).

The use of frameworks based on extended irreversible thermodynamics provides correspondences of the proposed model with experimental models characterized by matching reduced computational loads. In the context of extended irreversible thermodynamics (EIT), Chen and Yeh [30] proposed a phenomenological theory for MR fluids that connects the dynamics of flows to a non-equilibrium-state equation and naturally includes the elasticity of MR fluids. In this paper, to describe the mechanisms of energy transfer and dissipation, the Gibbs equation and the entropy inequality are used. Versaci and Palumbo [31] confirmed the association of the underlying shear flow and dilution behavior of the Herschel-Buckley plastic component from a known experimental model with elasto-viscoplastic generalization under the generalized standard materials.

Magnetic nanoparticles have the potential to be magnetic contrast agents in biomedical magnetic imaging. Their interference in cellular biological systems such as those of a cell (10–100 nm), virus (20–450 nm), protein (5–50 nm), or gene (2 nm wide-ranging and 10–100 nm) due to their size can be adjusted from a few nanometers to tens of nanometers [32]. Their existence in the biological systems under investigation may be determined using appropriate sensitive components (biosensors), which are attractive for magnetic bio-detection due to their high sensitivity, compact size, low power consumption, rapid response, and low cost [32].

The potential of magnetic nanoparticle internalization has been illustrated by different in vitro studies. Internalization of maghemite (Fe_2_O_3_) or magnetite (Fe_3_O_4_) nanoparticles by cells have been demonstrated for diverse cellular types [32,33,34]. Nanotechnology has the potential to improve the selectivity and efficacy of chemical, physical, and biological methods of killing cancer cells while reducing damage to noncancerous cells. Magnetic nanoparticles have also been used in numerous biomedical applications, such as hyperthermia treatment, radioimmunotherapy, and magnetic resonance imaging [34].

Nanotechnology can detect changes in a small number of cells due to their small size. It can distinguish between cancerous and normal cells. It can do these in the early stages of cancer when the cancer cells are just starting to divide, and thus when the disease is easier to treat. Nanotechnology may also make it easier to detect tumors in imaging tests. Tumor targeting is one of the main potential advantages of nanotechnology for cancer treatment. The ability to distinguish between malignant and nonmalignant cells and selectively eliminate malignant cells is critical to the purpose of nanotechnology in cancer treatment. Malignant and non-malignant cell differentiation procedures fall into two categories: passive and active targeting [35,36]. Nanoparticles coated with antibodies or other chemicals are likely to identify and stick to cancer cells. If the particles come into contact with cancer, they can be coated with compounds that send a signal [37]. Nanomaterials are increasingly being targeted at highly sensitive cancer cells, both actively and passively [38]. Cancer treatments can be made safer and more accurate with nanotechnology. Specially designed nanoparticles administer chemotherapy directly to tumors. Their small size enables them to transport drugs to hard-to-reach parts of the body. They only give drugs after they reach their destination. This prevents the drugs from causing damage to healthy tissue around the tumor, or other side effects as a result of injury.

The design, safety, and extraction of geothermal energy from deep subterranean areas are based on research into the interaction of fluids and heat in the surrounding deep fissured rock. To generate an unstable 3D model of fluid-heat coupling heat transfer in the surrounding fractured rock, fractured media and heat transfer hypotheses were used [39].

Mathematics and medical sciences are important disciplines that a person cannot do without, and they have an important relationship in almost all areas of life. For example, the doctor calculates the drug dose using medical equations. Mathematics also enables doctors to calculate the percentage of a patient’s dehydration and the amount of water that the patient needs, as well as the number of calories in the human body.

Other models of bioheat transfer are being constructed to account for the diverse nature of living tissues. The dynamic theory of thermal elasticity was combined with the non-Fourier thermal conductivity equation to perform the finite-elastic wave heat analysis in this study. In many of these applications, the use of the Cattaneo–Vernotte conduction model in place of Fourier’s law and/or the calculation of the temperature dependency of material parameters has improved predictions. The generalized models proposed by Lord and Shulman [40] and Green and Lindsay [41] are the first two common generalized models of thermoelectricity. Green and Naghdi suggested the following thermoelectricity generalization [42,43,44]. Their model is divided into three groups, known as thermoelectricity types I, II, and III. Tzou [45,46,47] suggested the thermoelastic model with phase lags.

The Moore-Gibson-Thompson (MGT) equation is famous for being a linear model of wave propagation in viscous thermodynamic fluids. The MGT equation is one of the most prominent models in the world of sound waves in terms of physics. For example, high-intensity ultrasound has been used in medical imaging, treatment, ultrasonic cleaning, and welding. The third-order differential equation, which is combined into the value of the dynamic properties of different fluids, gave rise to this concept. Quintanilla [48] has developed a new model of thermoelastic conduction (MGT thermoelectricity) based on the MGT equation. Quintanilla [49] also proposed a new two-degree thermoelastic model in which thermal conductivity is determined as the historical MGT version, which arose from the development of the Green-Naghdi Type III theory by adding the relaxation modulus. This theory began with a third-order differential equation created in the context of fluid mechanics. Since the introduction of the MGT theory [50,51,52,53,54,55,56,57,58,59,60], many researchers have devoted their efforts to studying this new model.

In the recent decade, many researchers have focused on using different hyperthermia approaches to apply distinct bioheat models in cancer therapy [52,53,54,55,56,57,58,59,60,61,62,63,64]. Numerical and analytical approaches have been used to solve mathematical models, and certain adjustments have been made. Analytical research of bioheat transfer is critical for a variety of medical applications, such as heat-driven cancer therapies. While bioheat transfer has been studied under a variety of conditions, there is relatively little research on bioheat transfer in a multilayered material such as skin. The description of several models that have been used to solve hyperthermia problems over the years is included in this study.

This work–study provides an analytical solution to the modified Pennes bioheat equation, which includes the MGT equation, in biological tissues as an infinite concentric spherical area during magnetic fluid hyperthermia. While bioheat transfer has been studied under a variety of conditions, there is relatively little research on bioheat transfer in a multilayered material such as the skin. in this study, the temperature distribution in the skin was calculated numerically, and the complete solution was determined using the interface temperature compatibility criteria and the heat flow compatibility criteria. This solution was intended to investigate the impact of heat generation during thermal treatment of a skin tumor represented as a spherical region. The temperature of the tumor was raised to roughly 42 °C for an hour or more during the hyperthermia.

The temperature responses of the generalized and classical MGT bioheat models were compared. The MGT bioheat models were generated from the constitutive MGT model and the Pennes bioheat equation, as well as from the classical Fourier heat transfer model. The model may take into consideration the effects of various therapeutic procedures such as cryotherapy, laser treatment, and other similar therapies, as well as physical processes such as transmission, blood perfusion, and metabolic activity. The model was used to study the effect of different physical factors on temperature profiles. This theoretical approach, as well as the quantitative data presented here, may help to improve our knowledge of bioheat transfer in layered structures such as the skin.

## 2. Mathematical MGT Bioheat Model

Fourier’s law is one of the most famous phenomenological models of mathematical physics, although it is not without flaws. The most well-known of its predictions is that thermal conductivity is a diffusion process in which temperature changes propagate at infinite rates, which means that thermal deflection made at one location in a solid medium is instantly detectable anywhere in the material. The law of heat conduction formulated by Fourier is as follows:

(1) q=−K∇T,

where q represents the heat flow, K denotes the thermal conductivity, and T is the local tissue temperature.

The Pennes model [14] was created to forecast heat transport in the human forearm. Because of its simplicity, the Pennes bioheat equation has been used in a variety of biological research projects, including therapeutic hyperthermia for cancer therapy. Pennes equation is stated in its most basic form as:

(2) $$\rho C_{p}\frac{\partial T}{\partial t}=-\nabla \cdot q+q_{p}+q_{m}-W_{b}\rho_{b}C_{b}\theta ,$$

where $C_{p}$ is the specific heat of the tissue, $W_{b}$ is the local tissue blood perfusion rate, $\rho_{b}$ is the density of blood, $C_{b}$ is the specific heat of the blood, $\theta =T-T_{a}$, $T_{a}$ is the arterial temperature, ρ is the density of the tissue, $q_{p}$ is the rate of energy deposition, and $q_{m}$ is the metabolism, which is often slight in contrast to the external power deposition phrase, $q_{p}$. The Pennes equation, Equation (2), assumes that heat transfer between blood arteries and surrounding tissue happens mostly through capillary walls, where blood velocity is very slow.

Various changes to the Pennes bioheat transfer (PBT) equation have been proposed throughout the years to address the inconsistencies. One of these changes is the modified heat flux formula that considers the slow propagation speed of thermal waves in a biological medium [65,66]. On the other hand, the literature has indicated that thermal activity in nonhomogeneous media needs a relaxation period to collect enough energy to move to the next element and that the relaxation time in biological tissues is significant [67].

Cattaneo extended Fourier’s law by adding the relaxation time, $\tau_{0},$ with regard to the vector of the heat flow as follows:

(3) $$q+\tau_{0}\frac{\partial q}{\partial t}=-K\nabla T.$$

The coefficient $\tau_{0}$ in Equation (3) represents the intrinsic relaxation time, which is the time it takes for heat transfer to move within the volume element once the temperature gradient is created. As a result, the new word “thermal inertia” was coined. The transfer of heat within the medium is not instantaneous under this variation of Fourier’s law but occurs through the diffusion of heat waves with a finite velocity, a process referred to as the “second sound” [32,48]. The material under consideration determines the value of $\tau_{0}$. It has been experimentally determined for a wide range of materials and has been shown to be very short, at the picosecond scale in most metals, but up to 100 s in some biological tissues [10,24].

A typical hyperbolic bioheat equation can be derived by combining Equation (1) with the PBT equation, Equation (2). To explain the effect of restricted heat propagation, which must be treated for more realistic settings, Liu et al. [25] developed a generalized thermal wave model of bioheat transmission derived from non-Fourier convective heat transfer in living tissues. The modified PBT equation may be written as follows:

(4) $$\rho C_{p}\left(\frac{\partial T}{\partial t}+\tau_{0}\frac{\partial^{2}T}{\partial t^{2}}\right)=\nabla \cdot \left(K\nabla T\right)+\left(1+\tau_{0}\frac{\partial }{\partial t}\right)\left(q_{p}+q_{m}\right)-W_{b}\rho_{b}C_{b}\left(\theta +\tau_{0}\frac{\partial \theta }{\partial t}\right).$$

Green and Naghdi [41] proposed an alternative structure for heat diffusion as a new theory of thermoelectricity. The modified Fourier’s law of the GN-III model is stated as follows [41]:

(5) $$q=-\left[K\nabla T\left(x,t\right)+K^{*}\nabla \vartheta \left(x,t\right)\right].$$

In this equation, the parameter $K^{*}>0$ is a material characteristic that is constant and is also known as the “rate of heat conductivity.” Furthermore, the scalar function ϑ fulfills $\dot{\vartheta }=T$. Quintanilla [48] formulated the following revised version of the suggested modified heat MGT equation after including the relaxation component in the Green-Naghdi type III model:

(6) $$\left(1+\tau_{0}\frac{\partial }{\partial t}\right)q=-\left[K\nabla T+K^{*}\nabla \vartheta \right].$$

Taking the derivative of time in Equation (6) yields:

(7) $$\left(1+\tau_{0}\frac{\partial }{\partial t}\right)\dot{q}=-\left(K\nabla \frac{\partial T}{\partial t}+K^{*}\nabla T\right).$$

A modified Pennes bioheat transfer equation based on the MGT equation is constructed by combining Equations (2) and (7) as follows:

(8) $$\begin{array}{c} \left(1+\tau_{0}\frac{\partial }{\partial t}\right)\left[\rho C_{p}\frac{\partial^{2}T}{\partial t^{2}}+W_{b}\rho_{b}C_{b}\frac{\partial \theta }{\partial t}-\frac{\partial q_{p}}{\partial t}-\frac{\partial q_{m}}{\partial t}\right]=\nabla \cdot \left(K\nabla \frac{\partial T}{\partial t}\right)+\nabla \cdot \left(K^{*}\nabla T\right) \end{array}.$$

By adding the temperature increase $\theta =T-T_{a}$ and keeping $T_{a}$ fixed, Equation (8) may be converted into the following equation:

(9) $$\begin{array}{c} \left(1+\tau_{0}\frac{\partial }{\partial t}\right)\left[\rho C_{p}\frac{\partial^{2}\theta }{\partial t^{2}}+W_{b}\rho_{b}C_{b}\frac{\partial \theta }{\partial t}-\frac{\partial q_{p}}{\partial t}-\frac{\partial q_{m}}{\partial t}\right]=\nabla \cdot \left(K\nabla \frac{\partial \theta }{\partial t}\right)+\nabla \cdot \left(K^{*}\nabla \theta \right) \end{array}.$$

## 3. Formulation of the Problem

Assuming that magnetic nanoparticles are present in abundance in the tumor but not in the surrounding healthy tissue, the directly applied field heats only the tumor. We also assume that the perfusion component, which represents the heat transfer to the blood, is proportional to the volumetric blood flow and the difference between the local tissue temperature and the arterial temperature. Since capillaries are generally more or less evenly distributed in the tissue layer, the blood perfusion is uniform across healthy and injured tissues.

**Figure 1:**
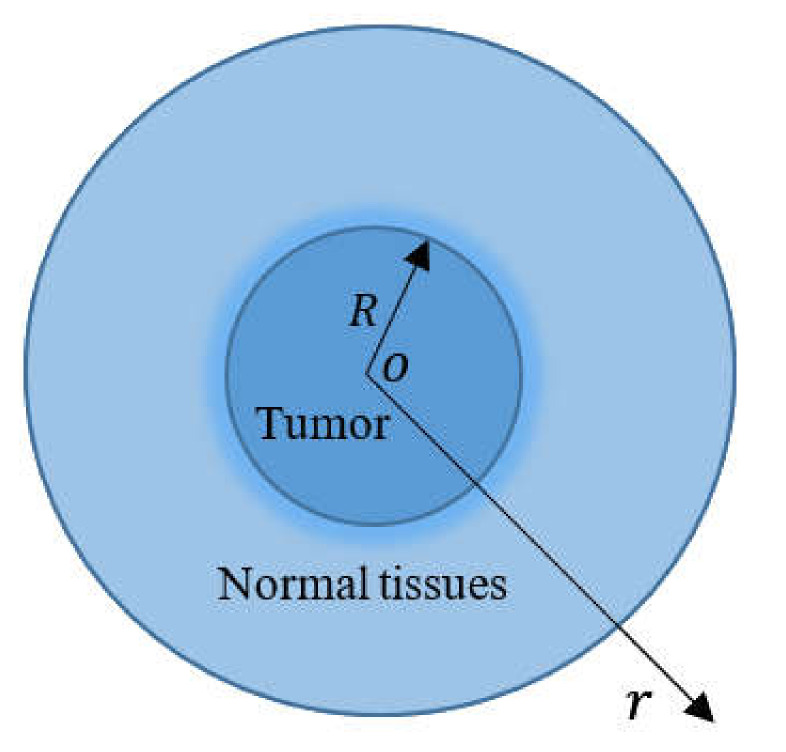
Model of a spherical tumor with a radius R implanted in normal tissue.

As seen in Figure 1, a small tumor is treated as a solid sphere with a radius R, and it becomes a heat source with a constant energy density, P, to excite an alternating magnetic field in the small tumor. With the origin used as the center of the sphere, we consider the spherical polar coordinates (r, ψ, ϕ). Heat travels evenly in the direction of the radius when t>0. The temperature change in the tumor (0≤r≤R) and in the normal tissues (R≤r≤∞) depends on the distance r from the center of the sphere and on time t. The field variables of the sphere under discussion become axially symmetric due to their axial symmetric shape, material properties, and loading conditions. As a result, the system of differential equations is simplified into a one-dimensional system, assuming a time-dependent metabolic heat source, $q_{m}=q_{0}e^{-t/t_{p}}$, in the tumor, and a constant in the normal tissues, whilst ignoring external heat sources, $q_{p}$. In Figure 1, the temperature is accumulated in an area concentric with the tumor and extending to the radius r.

The Pennes bioheat equation in the tumor and normal tissues with constant physiological parameters can be stated as follows.

For the tumor (0≤r≤R):

(10) $$\begin{array}{c} \left(1+\tau_{0}\frac{\partial }{\partial t}\right)\left[\rho_{1}C_{1}\frac{\partial^{2}\theta_{1}}{\partial t^{2}}+W_{b1}\rho_{b}C_{b}\frac{\partial \theta_{1}}{\partial t}+\frac{q_{0}}{t_{p}}e^{-t/t_{p}}\right]+P=\left(K_{1}\frac{\partial }{\partial t}+K_{1}^{*}\right) \end{array}\left(\frac{\partial^{2}\theta_{1}}{\partial r^{2}}+\frac{2}{r}\frac{\partial \theta_{1}}{\partial r}\right).\;$$

For the normal tissues (R≤r≤∞):

(11) $$\begin{array}{c} \left(1+\tau_{0}\frac{\partial }{\partial t}\right)\left[\rho_{2}C_{2}\frac{\partial^{2}\theta_{2}}{\partial t^{2}}+W_{b2}\rho_{b}C_{b}\frac{\partial \theta_{2}}{\partial t}\right]=\left(K_{2}\frac{\partial }{\partial t}+K_{2}^{*}\right) \end{array}\left(\frac{\partial^{2}\theta_{2}}{\partial r^{2}}+\frac{2}{r}\frac{\partial \theta_{2}}{\partial r}\right).$$

The initial conditions are considered as follows:

(12) $${\left.T\left(r,t\right)\right|}_{t=0}=T_{a},{\left.\frac{\partial T\left(r,t\right)}{\partial t}\right|}_{t=0}=0,{\left.\frac{\partial^{2}T\left(r,t\right)}{\partial t^{2}}\right|}_{t=0}=0.$$

We assume that the limit conditions of the problem at both ends satisfy the following equation:

(13) $$K_{1}{\left.\frac{\partial \theta_{1}\left(r,t\right)}{\partial r}\right|}_{r=R}=K_{2}{\left.\frac{\partial \theta_{2}\left(r,t\right)}{\partial r}\right|}_{r=R},\theta_{1}\left(R,t\right)=\theta_{2}\left(R,t\right).$$

## 4. Solution in the Laplace Transform Space

Using the Laplace transform method, which is described by the relation

(14) $$\bar{\theta }\left(r,s\right)=\int_{0}^{\infty }e^{-st}\theta \left(r,t\right)dt,\;\;\;\;\;Re\left(s\right)>0$$

to the Pennes bioheat Equations (10) and (11), we have

(15) $$\begin{array}{c} \frac{d^{2}{\bar{\theta }}_{1}}{dr^{2}}+\frac{2}{r}\frac{d{\bar{\theta }}_{1}}{dr}-m_{1}^{2}{\bar{\theta }}_{1}=F\left(s\right) \end{array}\;\mathrm{and}$$

(16) $$\begin{array}{c} \frac{d^{2}{\bar{\theta }}_{2}}{dr^{2}}+\frac{2}{r}\frac{d{\bar{\theta }}_{2}}{dr}-m_{2}^{2}{\bar{\theta }}_{2}=0 \end{array},$$

where s is the Laplace transform parameter for the time, and

$$\begin{array}{c} m_{1}^{2}=\begin{array}{c} \frac{\left(1+\tau_{0}s\right)\left(\rho_{1}C_{1}s^{2}+Wb_{1}\rho_{b}C_{b}s\right)}{\left(K_{1}s+K_{1}^{*}\right)},m_{2}^{2}=\frac{\left(1+\tau_{0}s\right)\left(\rho_{1}C_{1}s^{2}+W_{b2}\rho_{b}C_{b}s\right)}{\left(K_{1}s+K_{1}^{*}\right)},\mathrm{and} \end{array}\\ F\left(s\right)=\frac{q_{0}\left(1+\tau_{0}s\right)}{\left(K_{1}s+K_{1}^{*}\right)\left(t_{p}s+1\right)}+\frac{P}{\left(K_{1}s+K_{1}^{*}\right)}. \end{array}.$$

Equation (15) has a general solution that is limited as r→0 and is provided by:

(17) $$\begin{array}{c} {\bar{\theta }}_{1}\left(r,s\right)=- \end{array}\frac{F\left(s\right)}{m_{1}^{2}}+\frac{1}{\sqrt{r}}A_{1}I_{1/2}\left(m_{1}r\right),\;\;\;\;\;0\leq r\leq R.$$

The general solution of Equation (16), which is bounded by r→∞, is given by:

(18) $${\bar{\theta }}_{2}\left(r,s\right)=\frac{1}{\sqrt{r}}A_{2}K_{1/2}\left(m_{2}r\right),R\leq r\leq \infty .$$

The functions $I_{1/2}\left(m_{1}r\right)$ and $K_{1/2}\left(m_{2}r\right)$ are the modified Bessel function of the first and second types of order 1/2, respectively. The parameters $A_{1}$ and $A_{2}$ are constants that may be calculated based on the boundary conditions (13). In the Laplace transform domain, the boundary conditions (13) may be expressed as

(19) $$K_{1}{\left.\frac{\partial {\bar{\theta }}_{1}\left(r,s\right)}{\partial r}\right|}_{r=R}=K_{1}{\left.\frac{\partial {\bar{\theta }}_{2}\left(s,t\right)}{\partial r}\right|}_{r=R},{\bar{\theta }}_{1}\left(R,s\right)={\bar{\theta }}_{2}\left(R,s\right).$$

The boundary conditions provided by Equation (19) are utilized to get the constants $A_{1}$ and $A_{2}$.

A numerically reversed technique based on the Riemann sum approximation method is utilized to examine the numerical results for the ultimate solution of the temperature variation. Using this method, any function $\bar{\theta }\left(r,s\right)$ in the Laplace domain may be inverted into $\theta \left(r,t\right)$ in the time domain, as shown in [68]:

(20) θr,t=eλtt12Reθ¯r,λ+Re∑n=0N−1nθ¯r,λ+inπt,

where λ is an arbitrary real number higher than the real portions of all singularities of the function $\bar{f}\left(r,s\right)$, Re is the real part, and $i=\sqrt{-1}$. According to the numerical calculations, the value that fulfills the abovementioned connection is as high as λ≈4.7/t [46], which allows for quicker convergence.

## 5. Evaluation of the Thermal Damages

Thermal treatments need a precise prediction of thermal damage to skin tissues. One of the most essential aspects of hyperthermia cancer therapy is the assessment of burns. The techniques described by Mortiz and Henriques [69] can be adapted to the assessment of thermal damages caused by such radiation therapy or chemotherapy. To estimate skin temperatures, a numerical model based on Crank-Nicolson’s implicit computational technique is generally employed. An Arrhenius relationship is used to predict burn damage based on the skin temperature distribution [70,71], as follows:

(21) $$\bar{\Omega }\left(r,s\right)=\int_{0}^{t}Be^{-\left(\frac{E_{a}}{R_{g}}\right)/\bar{T}\left(r,s\right)}dt,$$

where $R_{g}=8.313\;J/\left(\mathrm{mol}K\right)$ represents the universal gas constant, $B=3.1\times 10^{98}\;s$ represents the factor of frequency, and $E_{a}=6.28\times 10^{5}\;J/\mathrm{mol}$ signifies the activation energy.

## 6. Numerical Results

We now present some numerical results to clarify our theoretical conclusions in the previous part and to show the impact of the blood perfusion rate and thermal relaxations on the temperature change. We used the MGT Pennes bioheat equation to examine heat transmission in normal and malignant tissues in the dermal regions of the human body via conduction and temperature-dependent perfusion. The epidermal layer of the skin is not perfused, while its deep tissue consists of the dermis and subcutaneous layers, with perfusion playing a key part in predicting temperature distribution changes. In this model, the role of oscillatory heat flow in predicting the spatial temperature distribution of normal and tumor tissues was investigated. We used the Mathematica software to calculate the numerical values.

The unregulated and duplicated development of tumor cells causes abnormal temperature fluctuations in the surrounding normal tissues. Local hyperthermia treatment, which involves delivering a focused beam of heat radiations to the tumor’s source, can destroy tumor cells. The constant use of heat may harm the normal tissue cells that surround the tumor. It is therefore critical to investigate the heat distribution in both normal and malignant tissue areas of the human body. A mathematical formula based on the Pennes bioheat equation with certain important parameters was used to evaluate the change in the tissue temperature.

Processes ranging from the nanoscale of cell membranes and organelles to the macroscale of the entire body determine heat transport in perfused tissues. The sizes of interest for most clinical applications range from the capillaries, with diameters of a few microns, to anatomical structures such as organs measuring several centimeters or more. In theory, by exposing a well-defined volume of the same composite to an alternating magnetic field, the density of power, P, absorbed by the composite may be calculated. The temperature rise may be measured as a function of time under adiabatic situations. However, provided that the particular magnetic losses and particle volume concentration are known, the power density, P, may be computed.

The current findings are for a tiny spherical tumor with a radius of R=0.002 m and a power density of $P=6.15\times 10^{6}\;W/m^{3}$ implanted in stretched muscular tissue. Also, the following are examples of values of different variables for human skin that were used in the numerical computations [72,73]:

$$\begin{array}{c} \left\{\rho_{1},\rho_{2}\right\}=\left\{1660,1000\right\}\left(\frac{\mathrm{kg}}{m^{3}}\right),\left\{C_{1},C_{2}\right\}=\left\{2540,3720\right\}\left(\frac{J}{\mathrm{kg}K}\right),T_{b}=T_{0}=37{}^{\circ}C,\\ \left\{K_{1},K_{2}\right\}=\left\{0.778,0.642\right\}\left(\frac{W}{\mathrm{mK}}\right),q_{0}=2900\left(\frac{W}{m^{3}}\right),\rho_{b}C_{b}=4.18\times 10^{6}\left(\frac{J}{m^{3}K}\right),\\ \left\{K_{1}^{*},K_{s}^{*}\right\}=\left\{17,16\right\}\left(\frac{W}{\mathrm{smK}}\right),\left\{W_{b1},W_{b2}\right\}=\left\{0.009,0.00018\right\}\left(\frac{1}{s}\right),\tau_{0}=2s. \end{array}\mathrm{and}$$

Living tissues have significant properties such as metabolic heat production and blood perfusion rates $W_{b1}$ and $W_{b2}$. According to the findings in [74,75], the metabolic heat generation and blood perfusion rates differ between tumors and normal tissue. On the other hand, Maenosono and Saita [76] utilized identical tumor and normal tissue values. Without accounting for the effects of blood perfusion and metabolism, Andrä W.et al. [73] calculated disparities in the breast temperature. The rate of metabolic heat production and blood perfusion is not unknown. This difference may have a significant influence on the temperature rise during hyperthermia treatment.

**Figure 2:**
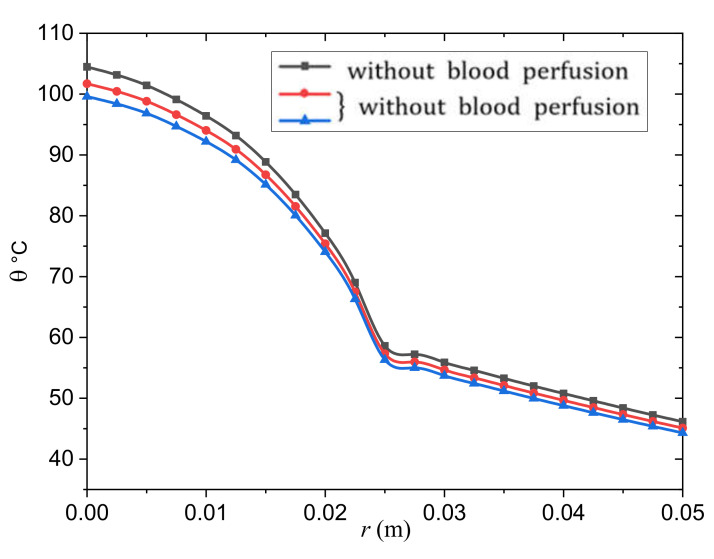
Effect of the blood perfusion $W_{b}$ change on the temperature change in the tumor and normal tissue.

As shown in Figure 2, the influence of the metabolic heat generation rate on the MGT non-Fourier bioheat transfer model is investigated in a wide range of skin distances 0≤0.02≤r≤0.05 m. The transient temperature increases in the tumor and normal tissues are shown in Figure 2 with and without the influence of the metabolic heat production rates and blood perfusion. In such situations, the perfusion rates $W_{b1}$ and $W_{b2}$ caused the temperature to decrease. In other words, blood perfusion rates serve as cooling mechanisms, as observed in [77]. As the temperature differential between tissue and blood grows, heat loss due to blood perfusion rises, slowing the rate of temperature rise θ, as illustrated in Figure 2. It is probable that the heat transfer rate in the tissues reaches a stable state as a result of the cooling impact of the blood perfusion.

The efficacy of local tumor heating is stronger for larger tumors than for smaller tumors when there is a considerably decreased perfusion in the tumor relative to the surrounding normal tissues. Thermal diffusion is responsible for this outcome. The bioheat equation may be used to show how the tumor size affects the temperature profile. The effect of a limited tumor volume on the tumor temperature rise θ is visible with regular blood flow in the tumor and in the surrounding normal tissues (Figure 2). If the blood flow through the tumor is lower than the blood flow through normal tissue, the temperature θ of the core tumor rises as the tumor size grows. These fascinating findings are straightforward to comprehend. Heat is distributed equally throughout the tumor and the neighboring tissues; and when the tumor blood flow is decreased, the temperature θ in the tumor is predicted to rise above that of the surrounding tissue. As a result, thermal diffusion will become a substantial mechanism of heat transfer out of the tumor, and it will be the only route of heat transfer out of the tumor in the limit of zero perfusion.

The temperature change in the tumor tissues (0≤r≤R) and in normal tissues (R≤r≤∞) depends on the distance, r, from the center of the sphere and time, t. This may be the reason for the sudden change in the spread of the heat wave or the presence of singular points in the figures.

**Figure 3:**
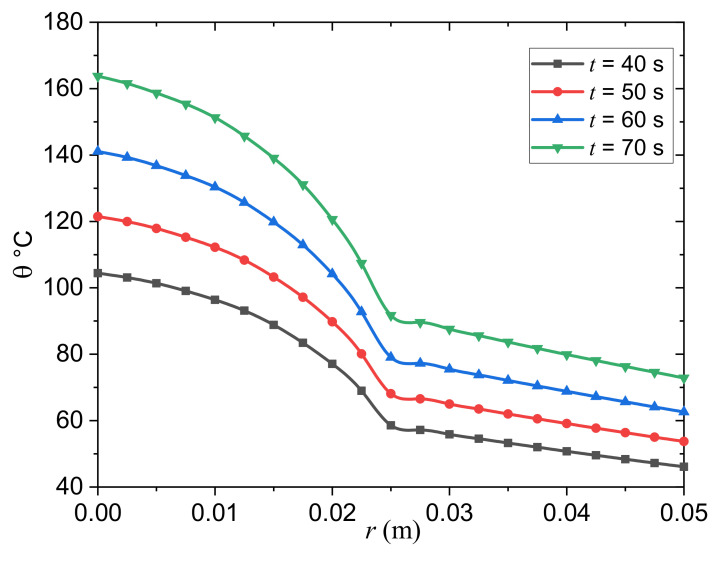
Effect of the instance time t on the temperature variation in the tumor and the normal tissues.

For various choices of the instance time, t (t=40, 50, 60 s), Figure 3 depicts the dynamical temperature, θ, rise in the tumor and normal tissues with regard to a wide range of the skin radius, r, $\left(0\leq 0.02\leq r\leq 0.05\right)$ m, and constant blood perfusion rates $W_{b1}$ and $W_{b2}$. The instance time, t, has a substantial influence on the temperature increment θ distribution in the tumor and normal tissues, as shown in Figure 3. The graphic shows that as the instance time parameter, t, is increased, the temperature θ rises. For the three examples, Figure 3 depicts the variation of the temperature distribution θ across the radial distance, r. As the radial distance, r, rises in the wave propagation direction, the thermal temperature θ in the figure falls. This indicates that heatwaves move at a slow rate within the medium, which is compatible with the physical side. This occurrence also highlights the importance of the modified PBT equation, which is based on the MGT equation, as well as its application as an alternative to traditional heat wave models that predict an infinite velocity.

**Figure 4:**
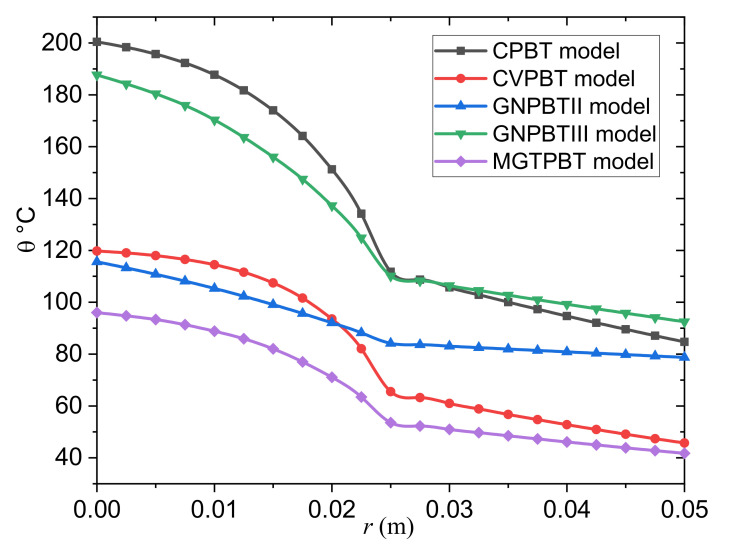
Temperature variation in the tumor and normal tissues for different models of the PBT equation.

As previously stated, the Moore–Gibson–Thompson Pennes bioheat transfer (MGTPBT) model assumes the energy equation for the blood subdomain in this study. The temperature distribution, θ, versus the distance, r, is examined in the last case, and several models of the PBT equation are evaluated at time t=50 s. The temperature variation is displayed in Figure 4.

The following models of the PBT equation can be obtained as special cases from the model derived in Equation (9):

- The classical Pennes bioheat transfer (CPBT) model can be obtained when we set $\tau_{0}=K^{*}=0$.
- The Cattaneo–Vernotte Pennes bioheat transfer (CVPBT) model can be obtained when we put $K^{*}=0$ and take $\tau_{0}>0$.
- The Pennes bioheat transfer model based on Green and Naghdi’s theory of type II (GNPBTII) can be obtained when the terms, including $\tau_{0}$ and K, are neglected.
- The Pennes bioheat transfer model based on Green and Naghdi’s theory of type III (GNPBTIII) can be obtained when the thermal relaxation time is neglected $\left(\tau_{0}=0\right)$.
- The new MGTPBT model is attained when $\tau_{0},K^{*}>0$.

From the graph, it can be seen that:

- The temperature distribution in the tumor and normal tissues is greatly influenced by the thermal factors $\tau_{0}$ and $K^{*}$.
- Including the relaxation coefficient, $\tau_{0},$ in the CVPBT and MGTPBT models may mean that the temperature decrease is slowed down.
- The predictions of the GNPBTIII and MGTPBT models are incompatible.
- The magnitude is larger in the case of the GNPBTIII model than in the case of the MGTPBT model, although the graph shows similar results for both models.
- The thermoelastic results of the GNPBTIII model differ significantly from the GNPBTII model due to energy losses in the case of the first model.
- In contrast to previous modified bioheat models, the results of the GNPBTIII model of thermoelasticity indicate convergence with the results of the conventional CPBT model, which do not fade in heat rapidly within the tumor and normal tissues, respectively.
- The profiles of the temperature differences between the MGTPBT and CVPBT models were compared. It is clear from the figure that the behavior and convergence of the results of both models are quite similar, with only slight differences in magnitude.
- The blood temperature distribution slightly differed between the MGTPBT and GNPBTII models.
- Heat wave propagation may realistically predict the temperature distribution in living tissues. The cooling function of the blood circulation keeps the tissue temperature from increasing but does not affect the speed of the thermal diffusion. According to this new hypothesis, the relaxation coefficient will become a new measure of the efficiency of the vital heat transfer in living tissues.
- Transferring thermal energy away from the interface is difficult to apply. As a result, the temperature gradually decreases. It indicates that by lowering the relaxation coefficient, the heat transfer capacity of the medium can be increased.

## 7. Conclusions

In this paper, the (MGT) equation is included in the modified Pennes bioheat conduction equation with a single relaxation time, and its impacts are shown in thermal treatment applications. The model considers blood circulation, metabolism, and other volumetric heat production processes. According to the research results, the non-Fourier Pennes bioheat model generates a higher temperature rise than the MGT Pennes bioheat model.

The conclusions from the main observations are as follows:

- The rate of change of blood perfusion has a significant effect on the transfer of bioheat in a tumor and normal tissue. Because the skin temperature is higher than the arterial temperature, the blood perfusion acts as a cooling agent. The perfusion rate is proportional to the amount of heat energy extracted from the blood.
- The results were influenced by the relaxation durations used and the perfusion of the blood. It is also clear that more experimental research is needed to determine the delay times more accurately.
- It was found that the presence of a thermal relaxation time in the biothermal conduction equation significantly affects the temperature spread in a tumor and in normal tissue over time. As a result, having a thermal relaxation time reduces the temperature drop as well as the tissue depth.
- This MGT Pennes bioheat model adds some additional dimensions to the investigation of transient heat transfer mechanisms in biological systems.
- The propagation of thermal waves may provide a realistic prediction of the temperature distribution in living tissue.
- In the case of the MGT Pennes biothermal model, the temperature spreads with a finite speed in the tumor and the normal tissue instead of an infinite speed in the classical model.
- The relaxation parameter can be proposed as a novel measure of bioheat transfer efficiency in living tissues in the revolutionary MGT Pennes bioheat model.
- The findings reported here may be of value for the design of many biomedical and biomechanical application areas, including in healthy and diseased tissues, as well as for the development of theoretical knowledge of bioheat transfer in spherical tissue architecture.

## Data Availability

The data presented in this study are available on request from the corresponding author. The data are not publicly available due to privacy reasons.
